# A Prospective Clinical Study on Postoperative Complications of Prostate Biopsy Following COVID-19 Infection at a Tertiary Hospital in Taizhou, China

**DOI:** 10.1155/cjid/6451174

**Published:** 2025-02-24

**Authors:** Dong-sheng Zhang, Yu-yi Chen, Jia-jia Zhu, Rong Wang, Liang-xue Sun

**Affiliations:** ^1^Department of General Surgery, The First People's Hospital of Jiande, Jiande, Zhejiang, China; ^2^Department of Urology, Taizhou Hospital of Zhejiang Province Affiliated to Wenzhou Medical University, Linhai, Zhejiang, China; ^3^Department of Operation, Taizhou Hospital of Zhejiang Province Affiliated to Wenzhou Medical University, Linhai, Zhejiang, China

**Keywords:** COVID-19 infection, postoperative complications, prostate biopsy

## Abstract

**Background:** Postoperative complications in individuals with a prior history of COVID-19 infection have been insufficiently investigated. This study is conducted to explore the postoperative complications of prostate biopsy in patients following a COVID-19 infection.

**Materials and Methods:** Data from individuals who underwent a prostate biopsy at a tertiary hospital in Taizhou city from 1 February to 15 November 2023 were collected, including a history of COVID-19 infection, a history of chronic disease, and postoperative complications of prostate biopsy.

**Results:** A total of 526 participants were enrolled in the study, with 325 individuals having a prior history of COVID-19 infection. The interval between infection and prostate biopsy was 29.25 ± 12.75 weeks, with a fluctuation range from 0.71 to 87.57 weeks. In individuals with a history of COVID-19 infection, 72 were asymptomatic, 110 experienced respiratory symptoms, and 145 had fever. In total, 198 patients reported postoperative complications, which showed no statistically significant difference with a history of COVID-19 infection (*p*=0.217). The top three reported postoperative complications were hematuria, perineal pain, and urinary retention, which tended not to be related to a history of COVID-19 infection (*p*=0.448, *p*=0.991, and *p*=0.277, respectively).

**Conclusion:** The incidence of postoperative complications of prostate biopsy in post-COVID-19 patients, who currently have no symptoms of COVID-19 infection, was comparable to patients with no history of COVID-19 infection. In clinical practice, for males with a history of controlled COVID-19 infection, the risk of postoperative complications from prostate biopsy should not be a major concern.

## 1. Introduction

SARS-CoV-2 is the infectious agent that leads to coronavirus disease 2019 (COVID-19). The COVID-19 pandemic has affected a large number of individuals, causing significant morbidity and mortality worldwide since December 2019 [1]. The global struggle against consecutive waves of COVID-19 persists, spurred by the emergence of viral variants [2].

To date, although limited perioperative outcomes have been reported, evidence suggests that surgical risks are significantly higher in patients with COVID-19 compared to non-COVID-19 individuals. In a matched cohort study carried out in Italy, individuals with COVID-19 demonstrated increased surgical mortality and complications in comparison to those without COVID-19. As a result, the study suggests that surgery should be delayed for individuals with COVID-19 if feasible [3]. In a cohort research conducted in Canada, individuals with COVID-19 undergoing surgery experienced an elevated 30-day postoperative mortality rate of 15.9% [4]. Perioperative COVID-19 infection exhibited a substantial association with a heightened risk of postoperative pulmonary complications, as affirmed by a systematic review and meta-analysis of observational studies [5]. During the COVID-19 pandemic, efforts to minimize exposure risks led to delayed diagnosis, resulting in a significant reduction in the number of prostate biopsies (*p* < 0.05). However, there was an increase in cases of advanced prostate cancer (pT3b: 11.2% vs. 25.6%; lymph node–positive: 14.8% vs. 46.1%) and metastatic prostate cancer (5.9% vs. 9.3%). Consequently, the overall cost associated with delayed diagnosis is expected to rise in the near future [6].

The SARS-CoV-2 virus or substances related to COVID-19 infection persisted in the body for an extended period. Patients experiencing COVID-19 showed enduring elevations in the frequency of activated CD14^+^ CD16^+^ monocytes and plasmacytoid dendritic cells, in comparison to control individuals, 8 months post-infection [7]. Moreover, some individuals exhibited a persistent increase in the levels of type I (IFNβ) and type III (IFNλ1) interferon 8 months after infection [7]. The research discovered an association between COVID-19 and a combination of IFNβ, pentraxin 3, IFNγ, IFNλ2/3, and IL-6, demonstrating an accuracy range of 78.5% to 81.6% [6]. Furthermore, in individuals who have recovered from COVID-19, there is a clonal turnover in the memory B cell pool that persists beyond 6 months of infection [8]. The SARS-CoV-2 virus has been detected in various human bodily fluids, including bronchoalveolar lavage, sputum, saliva, blood, urine, and feces [9].

Due to the prolonged presence of the SARS-CoV-2 virus and related substances in the body, there is reason to suspect that individuals who have previously been infected with COVID-19 and are currently asymptomatic may face a higher surgical risk compared to those without a history of COVID-19 infection. However, the results of individuals who undergo surgery after recovering from COVID-19 were less explored, particularly within the Chinese population. Hence, we carried out a prospective clinical study to evaluate the safety of patients with a history of COVID-19 undergoing prostate biopsy in Taizhou, China.

## 2. Materials and Methods

### 2.1. Study Design and Population

This investigation was conducted in the Department of Urology at Taizhou Hospital, one of the largest tertiary hospitals in Taizhou, China. The target population comprised individuals who underwent prostate biopsy in the Department of Urology from 1st February 2023 to 15th November 2023. The prostate biopsy was performed by the same urologist who has been providing urology services for over 20 years and has been qualified for prostate puncture for more than 5 years. Prior to biopsy, multiparametric MRI (mpMRI) of the prostate was conducted on a 3.0 T GE Signa HDx MR scanner (GE Healthcare, Milwaukee, USA), without the use of an intrarectal coil. The imaging was interpreted in accordance with the 2015 PI-RADS v2 guidelines, assigning a score ranging from 1 to 5 based on the likelihood of clinically significant prostate cancer [10]. Patients with abnormal digital rectal examination (DRE) and/or elevated prostate-specific antigen (PSA) levels (tPSA > 10 ng/mL or tPSA > 4 ng/mL with F/T < 0.15) underwent transperineal, ultrasound-guided cognitive targeted biopsies under local anesthesia. Lidocaine and ropivacaine were administered by the urologist near the scrotum and outside the prostate capsule. Systematic biopsies with at least 12 cores were performed using a biplane TRUS probe (Esaote, Transducer TRT33) and an 18-G disposable needle. During the perioperative period, prophylactic antibiotics, either cefuroxime axetil or levofloxacin, were administered, with one tablet given twice daily.

**Figure 1:**
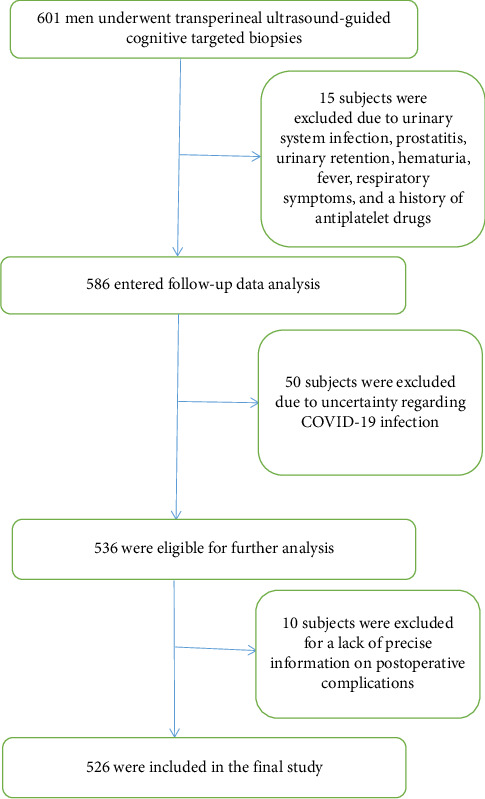
Flowchart of method for sample size determination.

Figure 1 depicts that initially, 601 patients were included in our study. Before the prostate biopsy, subjects with a history of COVID-19 infection provided the COVID-19 inspection certification at the hospital to determine the infection date. The infection date and the symptoms of COVID-19 infection, including asymptomatic infection, were recorded by our team. Subjects without suspicious COVID-19 infection symptoms (including fever, nasal congestion, hoarseness, headache, diarrhea, bellyache, and inappetence) since December 2021 (when the Chinese government lifted the strict epidemic control policy at that time) and a negative test for COVID-19, if conducted, were considered to be without COVID-19 infection. After the prostate biopsy, our team collected information on related postoperative complications, including urinary retention, hematuria, fever, irritation signs of the bladder, perineal pain, hematospermia, septicemia, phlebothrombosis, mortality, and unplanned readmission on the 7th day and the 30th day after the operation through telephone or face-to-face interviews. The exclusion criteria stipulated that subjects with urinary system infection, prostatitis, urinary retention, hematuria, fever, respiratory symptoms (including cough, nasal congestion, and hoarseness), a history of antiplatelet drugs before prostate biopsy, and those who were unclear about COVID-19 infection were not eligible for inclusion. Meanwhile, subjects without precise information on postoperative complications were excluded. Chronic diseases, including diabetes, hypertension, hyperlipidemia, chronic nephrosis, chronic obstructive pulmonary disease, cardiovascular disease, and cerebrovascular disease, along with smoking history and BMI, were documented in our study before the prostate biopsy. All methods were performed in accordance with the relevant guidelines and regulations. The Ethics Committee of Taizhou Hospital, Zhejiang Province, China, approved this study (approval number: KL20231212), and the study obtained informed consent from the participants. Figure 1 illustrates the screening procedure.

### 2.2. Statistical Analysis

The data in this study were analyzed using IBM SPSS Statistics 25.0 software. For numerical variables, descriptive statistics (mean ± standard deviation (SD)) were employed to characterize the sample. Categorical variables in the study were presented as percentages and counts. A *t*-test was used to analyze continuous variables. Qualitative variables were assessed using the *χ*^2^-test. Results were deemed statistically significant when the *p* value fell below 0.05.

## 3. Results

### 3.1. Study Population Characteristics

In total, 526 valid datasets were collected in our study. All subjects reported their postoperative complications. Among the 526 men, 201 were free of COVID-19 infection, while 325 were infected with COVID-19, and the interval between the COVID-19 infection date and the prostate biopsy date was 29.25 ± 12.75 weeks, with a fluctuation range from 0.71 to 87.57 weeks. All 325 of these patients reported their infection date, the symptoms of COVID-19 infection, and the therapeutic method. Only 1 patient reported having chronic nephrosis, and only 5 patients reported having chronic obstructive pulmonary disease. As indicated in Table 1, the demographic variables, including patients' age, BMI, and chronic diseases, showed no association with the history of COVID-19 infection. However, there was a statistically significant difference in smoking history concerning COVID-19 infection history.

### 3.2. Symptoms of COVID-19 Infection

Among 325 patients with a history of COVID-19 infection, 72 were asymptomatic, while 110 experienced respiratory symptoms, and 145 had a fever. The results are presented in Table 2.

### 3.3. Differences in Postoperative Complications Among Patients With a History of COVID-19 Infection

Table 3 reveals that a total of 198 patients reported postoperative complications following prostate biopsy, showing no statistically significant difference with COVID-19 infection history (*p*=0.217). No patients reported septicemia, phlebothrombosis, mortality, or unplanned readmission. The most prevalent postoperative complication was hematuria, accounting for 76.4% (162/212) of the total postoperative complications. The second and third most common postoperative complications were perineal pain and urinary retention, accounting for 9.9% (21/212) and 9.0% (19/212) of the total postoperative complications, respectively. The reported top three postoperative complications appeared to have no significant association with COVID-19 infection history (*p*=0.448, *p*=0.991, and *p*=0.277, respectively). Only a few cases of fever, irritation sign of the bladder, and hematospermia were reported (6, 3, and 1, respectively).

## 4. Discussion

### 4.1. Clinical Implications

To the best of our knowledge, this is the inaugural prospective clinical investigation revealing the correlation between a history of COVID-19 infection and postoperative complications for a specific operation. Our research could provide population-based empirical evidence to illuminate this matter. In our study, among the 526 patients, 198 reported postoperative complications. No statistically significant difference was observed between the history of COVID-19 infection and the incidence of postoperative complications.

Patients encountered higher risk of postoperative complications when contracted SARS-CoV-2 virus during the perioperative period. Comparing with asymptomatic COVID-19 patients, patients with symptomatic COVID-19 undergone emergency surgery had higher ICU admissions, prolonged length of stay in hospital, and decreased 90-day survival [11]. COVID-19 has been linked to elevated postoperative pulmonary complications and an increased risk of hemorrhage [12]. In a clinical study involving 211 patients undergoing colorectal cancer surgery, those operated on close to the time of COVID-19 infection faced elevated surgical risks. Conversely, surgery conducted more than a week after recovering from COVID-19 did not escalate the risk of postoperative complications [13].

There were limited retrospective studies to investigate COVID-19 infection history and postoperative complications. Deng et al.'s research identified an elevated risk of postoperative complications for elective surgery within 0 to 4 weeks following SARS-CoV-2 infection, an increased risk of postoperative pneumonia within 4 to 8 weeks after SARS-CoV-2 infection, and no heightened risk of complications 8 weeks after SARS-CoV-2 infection, through a retrospective collection of clinical data from 5479 patients [14]. Basing on a retrospective, case-control study in South India, incidence of postoperative complications, length of hospital stay, and 30-day mortality in patients with COVID-19 infection history undergoing clinical surgery were similar to patients without COVID-19 infection history [15]. This is consistent with the findings of our study. In 2020, the COVIDSurg collaborative group demonstrated that undergoing surgery more than 4 weeks after receiving a positive COVID-19 swab result was linked to a reduced risk of postoperative mortality [12].

Prostate cancer stands as a prominent contributor to cancer-related mortality in aging male patients, witnessing over 190,000 newly diagnosed cases annually in the United States and resulting in more than 33,000 deaths [16]. Prostate biopsies are vital for diagnosing and treating prostate cancer and stand as the predominant procedure administered by urologists, exceeding 2 million annually [17]. The indications for prostate biopsy involve an abnormal DRE and elevated levels of PSA, frequently assessed along with additional risk factors such as age, race, PSA velocity, and comorbidities [18]. Prostate biopsy is generally well received, and the probability of encountering significant complications is low. Nevertheless, frequently encountered minor complications, such as pain and bleeding, are commonly observed [19], and there has been an increasing trend in infectious complications over the years [20, 21]. Our study results similarly show that hematuria and pain are the most common complications. Additional complications linked to prostate biopsy encompass lower urinary tract symptoms (LUTS), urinary retention, and mortality [22]. In a study involving 8500 patients who underwent transperineal prostate biopsy, the incidence of clinical complications was 35.9%, yet only 1.5% of the patients required hospitalization. Urinary tract infection with fever was the most common reason for hospital admission, accounting for 33.4% of cases, with no patients developing sepsis. Both hospitalization rates and the frequency of emergency department visits were positively correlated with the number of biopsy needles used [23].

Our study revealed that patients undergoing prostate biopsy with no symptoms of current COVID-19 infection, even if infected with COVID-19 before, did not experience a higher risk of hematuria, urinary retention, and perineal pain when compared with patients with no history of COVID-19 infection. However, we still do not understand whether patients with a history of COVID-19 infection will experience increased postoperative complications in real-world settings. It needs to be further demonstrated in multicenter evidence-based studies.

### 4.2. Methodological Considerations

Our survey has some limitations. Firstly, this study was conducted solely in a hospital, which might not fully represent the entire target population. We intend to carry out large-scale multicenter studies to further address this issue. Secondly, patients with a history of COVID-19 infection primarily focused on December 2021 due to the Chinese government's policy regarding COVID-19. However, the study's considerable sample size lends a degree of credibility to its findings. Thirdly, the postoperative observation time is until the 30th day, which may omit complications occurring after that period. However, complications of prostate biopsy mainly occur within a month [22]. Finally, this study focuses on the specific procedure of prostate biopsy. Although its findings may not be applicable to the general population, they hold significant clinical relevance for the broader group of elderly male patients. Therefore, we firmly believe that this research has substantial value in guiding clinical practice.

In conclusion, the incidence of postoperative complications of prostate biopsy in post-COVID-19 patients, who currently have no symptoms of COVID-19 infection, was comparable to patients with no history of COVID-19 infection. In clinical practice, for males with a history of controlled COVID-19 infection, the risk of postoperative complications from prostate biopsy should not be a major concern.

## Figures and Tables

**Table 1:** Baseline characteristics of the patients.

Variables	All (*n* = 526) (%)	Infected (*n* = 325) (%)	Uninfected (*n* = 201) (%)	*χ* ^2^ or *T*	*p*
Age (y)	68.63 ± 8.63	68.13 ± 8.20	69.43 ± 9.26	1.685	0.093
BMI	23.91 ± 3.64	23.99 ± 3.85	23.79 ± 3.28	−0.607	0.544
Smoking history				13.47	< 0.001
Yes	141 (26.8)	69 (21.2)	72 (35.8)		
No	385 (73.2)	256 (78.7)	129 (64.2)		
Chronic diseases					
Diabetes				0.035	0.851
Yes	54 (10.3)	34 (10.5)	20 (10.0)		
No	472 (89.7)	291 (89.5)	181 (90.0)		
Hypertension				0.771	0.380
Yes	222 (42.2)	142 (43.7)	80 (39.8)		
No	304 (57.8)	183 (56.3)	121 (60.2)		
Hyperlipidemia				0.313	0.576
Yes	13 (2.5)	9 (2.8)	4 (2.0)		
No	513 (97.5)	316 (97.2)	197 (98.0)		
Cardiovascular disease and cerebrovascular disease				0.576	0.448
Yes	17 (3.2)	12 (3.7)	5 (2.5)		
No	509 (96.8)	313 (96.3)	196 (97.5)		
Chronic nephrosis				0.964	0.326
Yes	1 (0.3)	0 (0)			
No	324 (99.7)	201 (100)			
Chronic obstructive pulmonary disease				0.007	0.934
Yes	3 (0.9)	2 (1.0)			
No	322 (99.1)	199 (99.0)			

**Table 2:** Symptoms of COVID-19 among patients with a history of COVID-19 infection (*N* = 325).

Variables	*n* (%)
Symptom	
Asymptomatic infection	72 (22.2)
Respiratory symptom	110 (33.8)
Digestive tract symptom	3 (0.9)
Body ache	21 (6.5)
Fever	145 (44.62)
Fatigue	14 (4.3)

**Table 3:** Postoperative complications following prostate biopsy.

Variables	Number	Infected (*n* = 325) (%)	Uninfected (*n* = 201) (%)	*χ* ^2^	*p*
Total patients reporting postoperative complications	198	129 (65.2)	69 (34.8)	1.522	0.217
Total cases of postoperative complications	212	136 (64.2)	76 (35.8)		
Hematuria	162	104 (64.2)	58 (35.8)	0.576	0.448
Urinary retention	19	14 (73.7)	5 (26.3)	1.182	0.277
Fever	6	2 (33.3)	4 (66.7)	2.007	0.157
Irritation sign of the bladder	3	2 (66.7)	1 (33.3)	0.031	0.860
Perineal pain	21	13 (61.9)	8 (38.1)	0.001	0.991
Hematospermia	1	1 (100)	0 (0)	0.964	0.326

## Data Availability

The data from this study can be obtained with a reasonable request. Please contact Liang-xue Sun at 476311248@qq.com for data requests.
